# Endovascular treatment to improve outcomes for medium vessel occlusions: The ESCAPE-MeVO trial

**DOI:** 10.1177/17474930241262642

**Published:** 2024-06-20

**Authors:** Johanna M Ospel, Dar Dowlatshahi, Andrew Demchuk, David Volders, Markus Möhlenbruch, Shahid Nimjee, James Kennedy, Brian Buck, Jai Jai Shankar, Thomas C Booth, Mouhammad A Jumaa, Robert Fahed, Aravind Ganesh, Qiao Zhang, Craig Doram, Karla J Ryckborst, Michael D Hill, Mayank Goyal, the ESCAPE-MeVO investigators

**Affiliations:** 1 Department of Diagnostic Imaging, Foothills Medical Centre, University of Calgary, Calgary, AB, Canada; 2 Departments of Radiology and Clinical Neurosciences, Hotchkiss Brain Institute, University of Calgary and Foothills Medical Centre, Calgary, AB, Canada; 3 Department of Medicine, Ottawa Hospital Research Institute, University of Ottawa, Ottawa, ON, Canada; 4 Department of Diagnostic Radiology, Queen Elizabeth II Health Sciences Centre, Dalhousie University, Halifax, NS, Canada; 5 Department of Neuroradiology, University Hospital Heidelberg, Heidelberg, Germany; 6 Department of Neurosurgery, The Ohio State University Wexner Medical Center, Columbus, OH, USA; 7 Acute Multidisciplinary Imaging and Interventional Centre, Radcliffe Department of Medicine, University of Oxford, Oxford, UK; 8 Division of Neurology, Department of Medicine, University of Alberta, Edmonton, AB, Canada; 9 Max Rady College of Medicine, University of Manitoba, Winnipeg, MB, Canada; 10 Department of Neuroradiology, Ruskin Wing, King’s College Hospital NHS Foundation Trust, London, UK; 11 School of Biomedical Engineering & Imaging Sciences, King’s College London, London, UK; 12 Department of Neurology, ProMedica Toledo Hospital, Toledo, OH, USA

**Keywords:** Medium vessel occlusion, endovascular treatment, mechanical thrombectomy, acute ischemic stroke

## Abstract

**Rationale:**

Clinical outcomes in acute ischemic stroke due to medium vessel occlusion (MeVO) are often poor when treated with best medical management. Data from non-randomized studies suggest that endovascular treatment (EVT) may improve outcomes in MeVO stroke, but randomized data on potential benefits and risks are hitherto lacking. Thus, there is insufficient evidence to guide EVT decision-making in MeVO stroke.

**Aims:**

The primary aim of the ESCAPE-MeVO trial is to demonstrate that acute, rapid EVT in patients with acute ischemic stroke due to MeVO results in better clinical outcomes compared to best medical management. Secondary outcomes are to demonstrate the safety of EVT, its impact on self-reported health-related quality of life, and cost-effectiveness.

**Sample size estimates:**

Based on previously published data, we estimate a sample size of 500 subjects to achieve a power of 85% with a two-sided alpha of 0.05. To account for potential loss to follow-up, 530 subjects will be recruited.

**Methods and design:**

ESCAPE-MeVO is a multicenter, prospective, randomized, open-label study with blinded endpoint evaluation (PROBE design), clinicaltrials.gov: NCT05151172. Subjects with acute ischemic stroke due to MeVO meeting the trial eligibility criteria will be allocated in a 1:1 ratio to best medical care plus EVT versus best medical care only. Patients will be screened only at comprehensive stroke centers to determine if they are eligible for the trial, regardless of whether they were previously treated at a primary care center. Key eligibility criteria are (1) acute ischemic stroke due to MeVO that is clinically and technically eligible for EVT, (2) last-known well within the last 12 h, (3) National Institutes of Health Stroke Scale > 5 or 3–5 with disabling deficit, (4) high likelihood of salvageable tissue on non-invasive neuroimaging.

**Study outcomes:**

The primary outcome is the modified Rankin scale 90 days after randomization (shift analysis), whereby modified Rankin Score 5 and 6 will be collapsed into one category. Secondary outcomes include dichotomizations of the modified Rankin Score at 90 days, 24 h National Institutes of Health Stroke Score, difference between 24 h and baseline National Institutes of Health Stroke Score, mortality at 90 days, health-related quality of life (EQ-5D-5 L), Lawton scale of instrumental activities of daily living score, reperfusion quality (MeVO expanded Thrombolysis in Cerebral Infarction Score) and infarct volume at 24 h, and cost-effectiveness of endovascular recanalization. Safety outcomes include symptomatic and asymptomatic intracranial hemorrhage and procedural complications.

**Discussion:**

The ESCAPE-MeVO trial will demonstrate the effect of endovascular thrombectomy in addition to best medical management vis-à-vis best medical management in patients with acute ischemic stroke due to MeVO and provide data for evidence-based treatment decision-making in acute MeVO stroke.

**Data access statement:**

The raw data discussed in this mansucript will be made available by the corresponding author upon reasonable request.

## Rationale

In a pooled patient sample of stroke due to medium vessel occlusion (MeVO) from two prospective cohort studies, 50% of patients did not achieve excellent clinical outcome (modified Rankin Score (mRS) at 90 days with best medical management, including intravenous thrombolysis when indicated, and one-third were not functionally independent (mRS 0–2) at 90 days.^1^ Endovascular treatment (EVT) is a highly effective treatment for stroke due to large vessel occlusion. With both improvement in technique and development of novel devices, numerous non-randomized studies and few retrospective studies pooling data from cohort studies and randomized trials now suggest improved outcomes with EVT in stroke due to MeVO.^2–6^

However, there is a greater risk of harm related to iatrogenic vascular injury in the more distal circulation. Distal occlusions may also be more likely to recanalize with medical thrombolysis.^7^ Therefore, assuming there is a benefit for EVT, the magnitude of benefit is likely to be smaller than that seen in large vessel occlusion stroke. Thus, we seek to understand the balance between magnitude of effect and any safety signal when treating patients with stroke due to MeVO. The ESCAPE-MeVO trial aims to show that acute, rapid EVT in patients with acute ischemic stroke due to MeVO results in better clinical outcomes compared to best medical management.

## Methods

### Design

**Figure 1.**
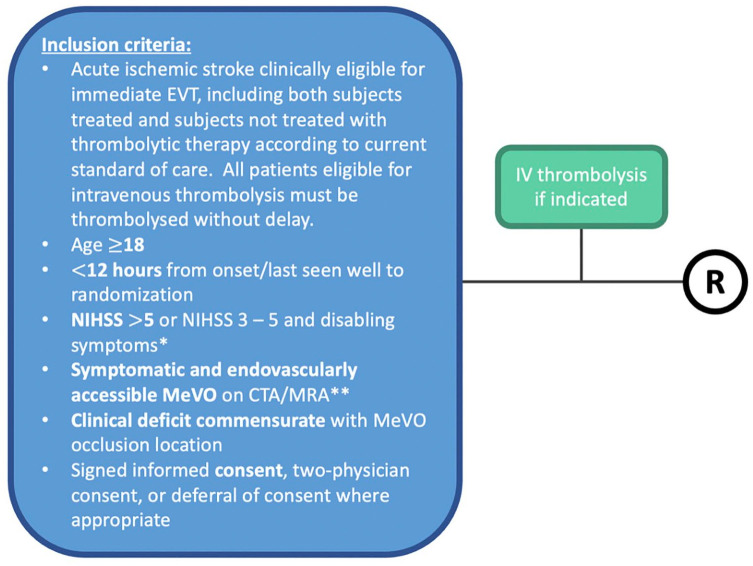
ESCAPE-MeVO enrollment scheme. CTP: CT perfusion; DWI: diffusion-weighted MRI (magnetic resonance imaging); h: hours; MRA: MR-angiography; NCCT: non-contrast CT; NIHSS: National Institutes of Health Stroke Scale; R: randomize. ^*^Disabling symptoms are defined by the local physicians. ^**^CT or MR angiography may be used to define MeVO location.

**Figure 2.**
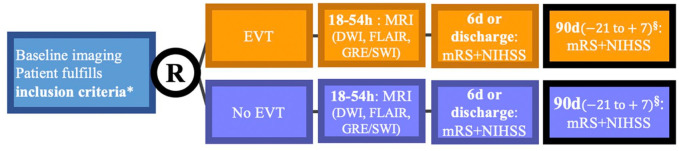
Patient flow in the ESCAPE-MeVO trial. D: days; DWI-MRI: diffusion-weighted MRI (magnetic resonance imaging); h: hours; MRA: MR-angiography; mRS: modified Rankin Scale; NCCT: non-contrast CT; NIHSS: National Institutes of Health Stroke Scale; R, randomization. For cases in which an MRI at 18–54 hours cannot be obtained (e.g. due to unavailability of an MR scanner, patient agitation etc.), an NCCT can be performed instead. ^*^Inclusion criteria are described in the following. ^§^In-person visit when possible. Telephone interview may be used in rare circumstances. Note that there are additional secondary outcomes not shown in the figure that are listed in the protocol.

ESCAPE-MeVO is a prospective, randomized, open label, parallel group design study with blinded outcome evaluation (PROBE design). Patients will be screened only at comprehensive stroke centers (i.e. EVT-capable) to determine eligibility, regardless of whether they were previously treated at a primary care center (i.e. non-EVT-capable). Both patients eligible and ineligible for intravenous thrombolysis will be included in the trial. If a patient is eligible for intravenous thrombolysis, it is expected that intravenous thrombolysis will be administered without delay (see Figure 1). Ethical approval will be obtained according to local and national regulations. All patients will provide informed consent or emergency consent according to relevant national standards. After randomization, patients undergo EVT or best medical management, according to the randomization assignment. The primary outcome is 90-day neurological disability scored on the modified Rankin Scale (see Figure 2).

### Patient population

Patients with acute ischemic stroke due to a clinically and technically EVT-eligible, symptomatic MeVO and reasonable likelihood of salvageable tissue on baseline neuroimaging will be included in the trial. Patients with intracranial hemorrhage, lack of salvageable brain tissue on baseline neuroimaging, severe co-morbid illnesses or severely limited life expectancy will not be eligible for the trial (see Table 1). Anatomical MeVO definitions are based on a prior publication^8^ and listed in Table 2.

**Table 1.** Inclusion and exclusion criteria in the ESCAPE-MeVO trial.

Inclusion criteria	
1	Acute ischemic stroke clinically eligible for immediate EVT, including both subjects treated and subjects not treated with thrombolytic therapy according to current standard of care. All patients eligible for intravenous thrombolysis must be thrombolyzed without delay
2	Age ⩾ 18 years at the date of randomization
3	Time from onset (or last-seen-well) to randomization < 12 h^a^
4	Disabling stroke defined as follows: (a) baseline National Institutes of Health Stroke Scale (NIHSS) score > 5 at the time of randomization (b) baseline NIHSS 3–5 with disabling deficit (e.g. hemianopia, aphasia, loss of hand function) as determined by the attending physician in context of the patient’s life situation at the time of randomization
5	Confirmed symptomatic and endovascularly treatable MeVO based on neurovascular non-invasive imaging (CTA or MRA)
6	Clinical deficit commensurate with MeVO location
7	Informed consent (process is site-specific)
Exclusion criteria	
1	Alberta Stroke Program Early CT Score ⩽ 5
2	Lack of salvageable brain tissue, defined as one of the following, depending on the imaging modality of the participating site: (a) NCCT + mCTA: Well demarcated hypodensity in the majority of the brain parenchyma supplied by the occluded vessel or absence of collaterals in the affected territory on the delayed phases of the mCTA (b) NCCT + (m)CTA/spCTA ± CTP: Lack of core:penumbra mismatch (if the CTP is uninterpretable, e.g. due to motion artifacts, apply exclusion criteria from 2(a). If spCTA rather than mCTA is performed, and the CTP was not performed, or was performed but is uninterpretable, score collaterals on spCTA and apply exclusion criteria from 2(a). If NCCT + mCTA + CTP are all performed, either the core:penumbra mismatch exclusion criteria from 2(b) or the exclusion criteria from 2(a) can be used) (c) MRI (DWI, MRA, ± MRP): Diffusion restriction in the majority of the brain parenchyma supplied by the occluded vessel or if MR perfusion is performed: lack of core:penumbra mismatch
3	Any evidence of intracranial hemorrhage on qualifying imaging
4	Patients living in a nursing home or requiring daily nursing care or assistance with activities of daily living
5	Patient has a major co-morbid illness, such as moderate or severe dementia, advanced cancer, advanced heart failure and so on, such that they are unlikely to be able to complete follow-up or they are unlikely to achieve the primary outcome due to the underlying illness (rather than the stroke or its treatment)
6	Pregnancy: female with positive urine or serum beta human chorionic gonadotropin (β-hCG) test
7	Participation in another clinical therapeutic intervention trial

EVT: endovascular treatment; MRA: MR-angiography; NCCT: non-contrast CT (computed tomography); CTP: CT perfusion; MRI: magnetic resonance imaging; DWI: diffusion-weighted MRI; MRP: MR perfusion; OR: odds ratio; NIHSS: National Institutes of Health Stroke Scale; mCTA: multiphase CT angiography; spCTA: single phase CT angiography.

a This choice was made based on time-windows used previously in the ESCAPE, ESCAPE-NA1 and ESCAPE-NEXT trials.

**Table 2.** Anatomical definitions of different MeVO locations used in the ESCAPE-MeVO trial.

MeVO location	Anatomical definition
Proximal M2 segment of the middle cerebral artery	Middle cerebral artery segments up to 1 cm distal to the main middle cerebral artery bifurcation/trifurcation
Distal M2 segment of the middle cerebral artery	Middle cerebral artery segments > 1 cm distal to the main middle cerebral artery bifurcation/trifurcation to the circular sulcus of the insula
M3 segment of the middle cerebral artery^a^	From the circular sulcus of the insula to the external/superior surface of the Sylvian fissure. Note that the distinction between distal M2 and M3 occlusions can be challenging. In general, the distal M2 segment has a more vertical course in the coronal plane, while the M3 segment has a more horizontal course, as it extends from the circular sulcus laterally to the cortical surface. This can be helpful in ambiguous cases
A2 segment of the anterior cerebral artery	From the origin of the anterior communicating artery to the origin of the callosomarginal artery
A3 segment of the anterior cerebral artery	From the origin of the callosomarginal artery to the artery’s posterior turn above the corpus callosum
P2 segment of the posterior cerebral artery	From the origin of the posterior communicating artery to point of entrance in the quadrigeminal cistern
P3 segment of the posterior cerebral artery	Segment within the quadrigeminal cistern

a Note that a non-dominant third-order middle cerebral artery third-order branch proximal to the circular sulcus of the insula will also be considered an M3 occlusion.

### Randomization

Treatment will be assigned using 1:1 randomization (best medical care control: EVT intervention) centrally, using a web-based algorithm. Randomization assignment will be fully concealed by having dynamic real-time allocation, based upon random number generation. The treatment will be unblinded (open-label). To minimize differences between the two treatment arms of the trial other than the investigational treatment, a randomized minimization procedure will occur on five baseline prognostic variables, namely age, sex, baseline NIHSS, occlusion location (anterior vs middle vs posterior cerebral artery), and site (minimal sufficient balance).^9^

### Intervention

Patients will be randomized to receive routine medical stroke care (control group) or to EVT plus medical care (intervention group). EVT will be performed using any Solitaire stent-retriever device (Solitaire X, Medtronic) as the first-line approach. Any combination of additional devices (e.g. aspiration and balloon guide catheters) may be used. In case the first-line attempt is unsuccessful, other stent-retrievers or aspiration catheters may also be used at the discretion of the neurointerventionalist. The use of general anesthesia versus conscious sedation or local anesthesia is considered a local decision according to the judgment of the local team. We expect that access to more distal parts of the intracranial circulation may require greater use of general anesthesia to allow for a completely still patient as compared to EVT for large vessel occlusion stroke.

Medical care is expected to apply to all patients and should include stroke unit care, early rehabilitation, investigations for stroke mechanism, stroke prevention treatment, and vascular risk reduction.

### Primary outcomes

The primary outcome is neurological disability as measured by the modified Rankin scale (mRS) 90 days after randomization.

### Secondary outcomes

Secondary outcomes are excellent outcome (mRS 0–1) at 90 days, good outcome (mRS 0–2) at 90 days, 24 h NIHSS, difference between 24 h and baseline NIHSS, mortality at 90 days, the Lawton index for instrumental activities of daily living at 90 days, health-related quality of life (EQ-5D-5 L) at 90 days and 1 year, final reperfusion quality (measured using the MeVO eTICI scale^2^), infarct volume at 24 h (measured in mL by manual planimetry), mRS at 1 year.

### Safety outcomes

Safety outcomes are symptomatic intracranial hemorrhage (defined as new intracranial hemorrhage with NIHSS decline ⩾ 2 points with the hemorrhage judged to be the most important cause of clinical worsening, alternative definitions^10^ will be applied in sensitivity analyses), and radiological hemorrhage (based on the Heidelberg criteria)^10^ on 24 h imaging, iatrogenic vessel perforation and dissection, major extracranial bleeding and access site neuropathy.

### Data monitoring body

The safety of the trial is overseen by an internal Clinical Events Committee and an independent Data Safety and Monitoring Committee (DSMC). Responsibilities and workflows in the DSMC are documented in a DSMC charter. The DSMC performed a safety review of the first 100 patients enrolled, and will conduct a formal safety analysis, futility and efficacy interim analysis after 250 patients have completed their 90-day visit (expected in the second quarter of 2024).

### Sample size estimates

The minimal clinically important difference for good outcome in acute stroke treatment has been identified at 5% in expert surveys.^11,12^ Based on data from prospective cohort studies,^1^ we estimated proportions of mRS 0/1/2/3/4/5-6 to be 34%/28%/11%/8%/7%/12% in the EVT arm and 24%/26%/8%/12%/18%/12% in the control arm. This results in a required sample size of 500 subjects to achieve a power of 85% with a two-sided alpha of 0.0398. To account for potential loss to follow-up (5%), the sample size was inflated to 530 subjects.

### Statistical analysis

The primary population for analysis will be the intention to treat population (as randomized) defined according to randomization assignment. The primary outcome (ordinal mRS at 90 days with mRS 5 and 6 collapsed into a single category) will be analyzed using a multivariable proportional odds model, adjusting for the five variables used in the minimization algorithm. A Wald test will be used to assess the hypothesis that the common odds ratio for an improvement in one or more points on the mRS scale is greater than 1. The primary analysis will be supported by an assessment of the unadjusted odds of achieving the primary outcome using an unadjusted comparison of proportions with the Wilcoxon rank sum test. The formal null and alternative hypotheses are as follows:

H_0_: The common odds ratio comparing treatment to control for improvement in one or more points on the mRS scale at 90 days, where categories 5 and 6 are collapsed, is not different from 1.
H_A_: The common odds ratio comparing treatment to control for improvement in one or more points on the mRS scale scored at 90 days, where categories 5 and 6 are collapsed, is greater than 1.

Pre-specified subgroup analyses will be performed stratified by intravenous thrombolysis and other key variables. Further details of the analysis plan are documented in a Statistical Analysis Plan that will be finalized prior to database lock.

## Study organization and funding

ESCAPE-MeVO is organized as an academic investigator-initiated trial. It is funded by grants to the University of Calgary from the Canadian Institutes of Health Research, a Canadian federal public funding body and by an unrestricted grant from Medtronic LLC, an industry partner. The executive committee is centered at the Calgary Stroke Program (Departments of Clinical Neurosciences, Radiology, Community Health Sciences, University of Calgary). Co-investigators and collaborators at sites in Canada, the United States, Europe, and the United Kingdom with long-standing experience in endovascular stroke management enroll patients at their sites. A steering committee manages the day-to-day activities of the trial.

## Discussion

Clinical outcomes in MeVO stroke are frequently poor, and EVT is a powerful treatment that can markedly improve outcomes in acute ischemic stroke due to large vessel occlusion. There is empirical evidence and non-randomized data supporting the use of EVT to treat not only large, but also MeVO stroke. Surveys suggest that many neurointerventionalists already routinely perform EVT for MeVO stroke, particularly if patients are ineligible for, or do not respond to, intravenous thrombolysis.^8^ However, EVT is not without risks, and at the same time, the margin of potential EVT benefit is likely to be smaller compared to stroke due to large vessel occlusion because the clinical course with best medical management is more favorable. It may well be that some, but not all, stroke patients with MeVO benefit from EVT. In addition to ESCAPE-MeVO, several other ongoing trials investigate the benefit of EVT in stroke due to MeVO (DISCOUNT:NCT05030142, DISTAL:NCT05029414, DISTALS:NCT05030142 and FRONTIER-AP:ACTRN12621001746820p). Together, these trials will not only answer the question whether EVT improves outcome in MeVO stroke but also provide data on whether some patient subgroups benefit more than others, on the time window in which MeVO EVT should be considered, safety and efficacy of different MeVO EVT techniques, and other important questions surrounding management of acute MeVO stroke.

## Conclusion

The ESCAPE-MeVO trial will demonstrate the effect of endovascular recanalization vis-à-vis best medical management in patients with acute ischemic stroke due to MeVO and provide data for evidence-based treatment decision-making in acute MeVO stroke.
